# Facile High Throughput Wet-Chemical Synthesis Approach Using a Microfluidic-Based Composition and Temperature Controlling Platform

**DOI:** 10.3389/fchem.2020.579828

**Published:** 2020-11-02

**Authors:** Yang Hu, Bin Liu, Yating Wu, Ming Li, Xiaorui Liu, Jia Ding, Xiaopeng Han, Yida Deng, Wenbin Hu, Cheng Zhong

**Affiliations:** ^1^State Key Laboratory of Metal Matrix Composites, School of Materials Science and Engineering, Shanghai Jiao Tong University, Shanghai, China; ^2^Key Laboratory of Advanced Ceramics and Machining Technology (Ministry of Education), School of Materials Science and Engineering, Tianjin University, Tianjin, China; ^3^Tianjin Key Laboratory of Composite and Functional Materials, School of Materials Science and Engineering, Tianjin University, Tianjin, China

**Keywords:** microfluidic, composition, temperature, high throughput, wet-chemical synthesis

## Abstract

The wet-chemical technique has been widely applied in material synthesis. In recent years, high throughput (HT) technique has shown its potential in parallel synthesis and the investigation of synthesis parameters. However, traditional ways of HT parallel synthesis require costly equipment and complex operating procedures, restricting their further applications. In this paper, we prepared a cost-effective and timesaving microfluidic-based composition and temperature controlling platform to carry out HT wet-chemical synthesis in a facile and automated workflow. The platform uses a microfluidic chip to generate 20–level concentration gradients of the two reagents and uses 100–channel reactor arrays for wet-chemical synthesis with 5–level temperature gradients. Scanning electron microscopy (SEM) and energy dispersive spectroscopy (EDS) were applied to characterize Co–Ni bimetallic powder materials synthesized under 100 different reaction conditions. X-ray photoelectron spectroscopy (XPS) was conducted to confirm the oxidation state of the products. This platform not only enables one-step determination of the minimum reaction temperature required for a wet-chemical system but also provides a significant increase in efficiency compared with the traditional wet-chemical approach. The microfluidic-based composition and temperature controlling platform shows promise in facile, efficient, and low-cost HT wet-chemical synthesis of materials.

## Introduction

Among the existing methods of chemical synthesis, the wet-chemical technique has been widely applied due to its versatility and flexibility (Yoshimura and Byrappa, 2008; Danks et al., 2016). The wet-chemical technique can be used to prepare numerous materials, ranging from structural materials such as ceramics (Sato, 2010; Palmero, 2015; Huang et al., 2017) and alloys (Dong et al., 2017; Wang et al., 2019), to functional materials such as energy materials (Ye et al., 2018; Chen et al., 2019; Guo et al., 2020; Song et al., 2020; Wu et al., 2020), optical materials (Rachna, 2013; Wang et al., 2017; Li et al., 2018), magnetic materials (Nakahara et al., 2010; Chen et al., 2013; Yang et al., 2017), and catalytic materials (Zhong et al., 2014; Qu et al., 2016; Ding et al., 2019, 2020). Researchers have put great effort into tuning the wet-chemical synthesis parameters like reagent concentration, temperature, reaction time, acidity (or pH) toward optimal reaction conditions for desirable product morphology, and characteristics (Huang et al., 2012; Jean et al., 2012; Zheng et al., 2018; Xu et al., 2019; Yan et al., 2020). However, the traditional wet-chemical synthesis approach cannot achieve large-scale investigations of synthesis parameters in a single experiment. For example, when we need to investigate the parameter of reagent concentration, the samples can only be prepared one after another. And if we need to investigate the parameter of reaction temperature simultaneously, the preparation time needs to be multiplied by the number of temperatures. Thus, investigating only one parameter with the traditional approach may still lead to multiple repeated operating procedures, let alone numerous parameters. As a result, the traditional wet-chemical synthesis approach is impractical or even infeasible in large-scale parameter investigations due to the high cost of time and manpower (Stock, 2010). Therefore, developing a new wet-chemical reaction system toward accelerated and automated parallel synthesis has become an urgent task.

Inspired by the pharmaceutical industry, the combinatorial method or high throughput (HT) strategy has been considered as a milestone in advancing the discovery of materials (Takeuchi et al., 2005). The HT strategy enables systematic and parallel investigation of numerous parameters, thus providing better understandings of how these parameters contribute to material synthesis (Bauer and Stock, 2007). In recent years, researchers have made progress in achieving HT synthesis under wet-chemical conditions. Moliner et al. prepared an automated HT platform for hydrothermal synthesis of zeolites (Moliner et al., 2005). Stock et al. developed an HT hydrothermal method allowing simultaneous investigation of up to 48 reaction mixtures of phosphonate-based compounds (Stock and Bein, 2004). However, the automated reagent injection processes and the reagent concentration control processes of these works require complex machines such as robotic systems and solid-dosing stations, leading to high-cost and complicated operating procedures.

Microfluidic technology stems from the efforts to dispense liquids in the range of nano- and subnanolitre (Haeberle and Zengerle, 2007). Microfluidic systems are suitable for large-scale automation and parallelization of chemistry and biology procedures, enabling multiple experiments to perform simultaneously while consuming little reagent (Squires and Quake, 2005). Now, microfluidic technology has been applied in medical diagnosis (Yager et al., 2006), cell biology (Brouzes et al., 2009), and drug discovery (Pihl et al., 2005; Dittrich and Manz, 2006). The applications of microfluidic technologies in HT chemical synthesis have also been reported these years. Liu et al. fabricated a co-flow microfluidic nanoprecipitation device out of glass capillaries toward HT synthesis of homogeneous nanoparticles (Liu et al., 2015). Wu et al. synthesized hierarchical metal–organic framework (MOF) nanosheet microcapsules produced on a large scale with a continuous droplet microfluidic approach (Wu et al., 2019). Feng et al. used a two-stage microfluidic chip to synthesize core-shell hybrid nanoparticles (Feng et al., 2015). In these works, the authors successfully obtained nanoparticles or droplets with different sizes and properties by varying flow rates of the fluids, thus providing solutions for controllable HT synthesis inside the microchannels. When facing the need for HT wet-chemical synthesis and parameter investigation, it is highly preferred that the parameters of reagent concentration and reaction temperature can be varied simultaneously. However, to our best knowledge, a reaction system which can meet such demands has not been reported. Therefore, a new wet-chemical reaction system enabling simultaneous investigation of numerous parameter combinations in a single experiment is highly necessary.

In the present work, we prepared a microfluidic-based composition and temperature controlling platform for facile HT wet-chemical material synthesis. The platform is aimed at investigating the synthesis parameters of reagent concentration and reaction temperature effectively and automatically without using robotic machines. Based on repeated splitting, mixing, and recombination of laminar flow fluids, certain types of microfluidic devices can serve as concentration gradient generators (Jeon et al., 2000). A group of positive temperature coefficient (PTC) heating plates serve as temperature gradient generators. By combining a microfluidic-based concentration gradient generator with a temperature gradient generator, a considerable amount of parameter combinations can be investigated simultaneously in a single experiment. Our microfluidic-based composition and temperature controlling platform provides significantly higher efficiency than the traditional approach. First, reagents with 20 different concentrations can be prepared in parallel instead of serially. Second, the process of reagent injection can be performed automatically instead of manually. Third, reactions under 5 different temperatures can be held simultaneously instead of one after another. By varying the parameters of reagent concentration and reaction temperature, the wet-chemical synthesis of Co–Ni binary powder materials was carried out in 100–channel reactor arrays. After the reaction, the minimum temperature required for the reaction system were determined directly by optical observation. Moreover, the scanning electron microscope (SEM) characterization results confirmed the successful synthesis of 60 different powder materials at 170, 200, and 230°C with a particle size of 0.5–2 μm. The energy dispersive spectrometer (EDS) analysis showed that the weight ratios of Ni to Co of the samples show a gradient pattern in general as expected. The X-ray photoelectron spectroscopy (XPS) analysis showed that the products contain Co^2+^, Co^3+^, Ni^2+^, metallic cobalt, and metallic nickel.

## Materials and Methods

### Construction of the Microfluidic-Based Composition and Temperature Controlling Platform

The microfluidic-based composition and temperature controlling platform consists of three functional regions, including the microfluidic region, the peristaltic pump region, and the reactor arrays region.

**Figure 1:**
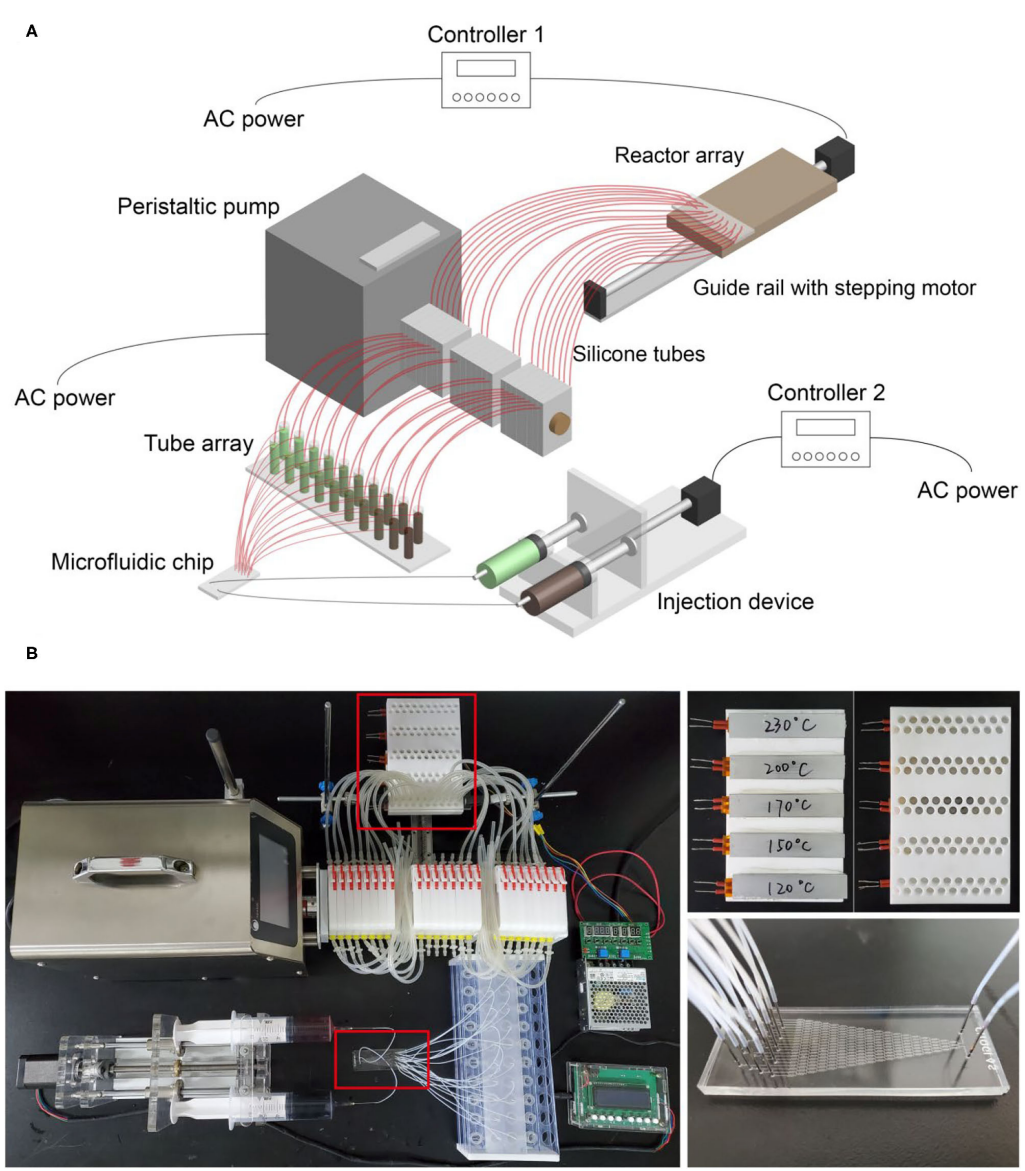
**(A)** Schematic diagram of the microfluidic-based composition and temperature controlling platform; **(B)** photo of the platform. The small photos on the right show details of the reactor arrays and the microfluidic chip.

In the microfluidic region, two 50 mL syringes filled with different reagents are fixed on an automated injection device driven by a stepping motor which is connected to controller 2 (See Figure 1A). The two inlets of the microfluidic chip are connected to the two syringes by polytetrafluoroethylene (PTFE) tubes with an inner diameter of 0.5 mm, while the 20 outlets of the microfluidic chip are connected to a tube array with 20 centrifuge tubes (4 mL). At the conjunction of the PTFE tubes and the microfluidic chip, steel needles with an outer diameter of 0.7 mm serve as the adapters to prevent leakage. When the injection device is running, the two syringes are squeezed at a constant speed (the speed can be adjusted using controller 2). Meanwhile, the reagents flow through the fluid channels of the chip, generating 20–level concentration gradients and finally flow into the 20 centrifuge tubes.

In the peristaltic pump region, the core component is the peristaltic pump which can transport multichannel fluids from one side to the other side in a controllable flow rate. Twenty silicone tubes pass through the pump with one end connected to the centrifuge tubes and the other end connected to the reactor arrays region. When the peristaltic pump is running, the reagents in 20 centrifuge tubes can be transported to the reactor arrays simultaneously with an identical flow rate. We use the peristaltic pump not only to enable automated operation but to make sure an identical volume of reagents can be transported to each reactor of the reactor arrays.

In the reactor arrays region, the reactor arrays are fixed on a guide rail driven by another stepping motor which is connected to controller 1 (See Figure 1A). A transparent board with 20 holes is clamped by two iron stands horizontally, and the silicone tubes are fixed by the holes. In this way, the fluids can go through the silicone tubes and flow into designated reactors directly by gravity. By matching the settings of controller 1 and the peristaltic pump, automatic reagent injection into the reactor arrays can be achieved. Initially, one group of the reactor arrays (20 reactors) is vertically aligned with the 20 silicone tubes. Step 1, the peristaltic pump is set to run, and reagents with 20 different compositions fill into the 20 reactors. Step 2, controller 1 is set to run, and the reactor arrays move along the direction of the guide rail for a distance identical to that of two reactor groups. In this way, another group of the reactor arrays is aligned with the silicone tubes. After repeating step 1 and step 2 for five times, all five groups of the reactor arrays (100 reactors) are filled with desired reagents.

The photo of the platform is shown in Figure 1B. The whole operating process was recorded and provided as Supplementary Videos 1, 2 to show how we achieved automatic operation using the microfluidic-based composition and temperature controlling platform (here we used red and blue ink for substitution of reagents to show the concentration gradients more clearly).

### Details of the Microfluidic Chip and the Reactor Arrays

**Figure 2:**
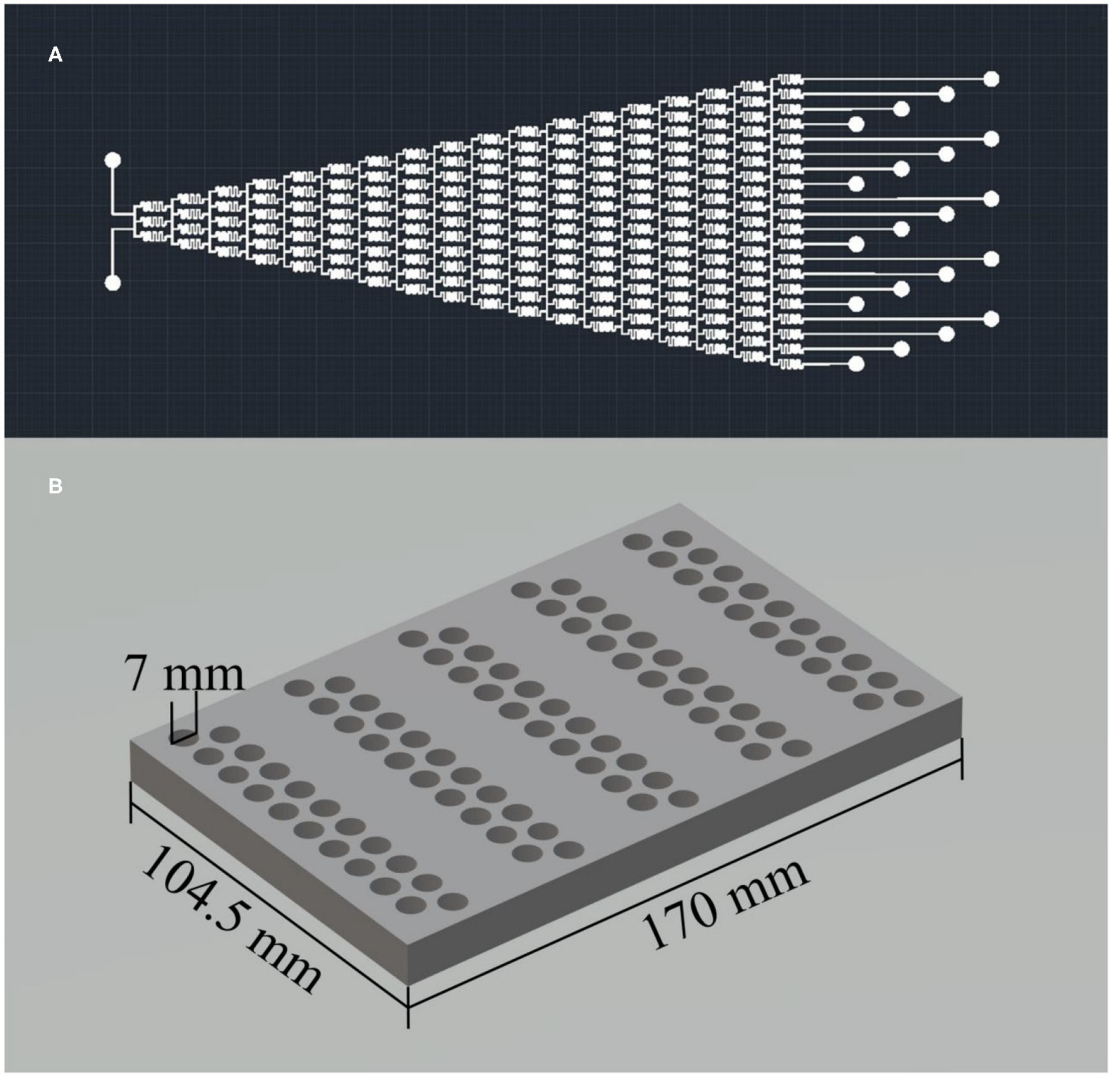
**(A)** The design pattern of the microfluidic chip and **(B)** the 3D model of the reactor arrays.

The microfluidic chip serves as the concentration gradient generator and is the core component of the platform. It was manufactured by soft lithography using polydimethylsiloxane (PDMS) as the material based on a silicon mold (shown in Supplementary Figure 1). Figure 2A shows the design pattern of the microfluidic chip, which has the feature of two inlets and 20 outlets with a diameter of 0.5 mm. The flow channels inside the chip show a Christmas-tree-type structure with a cross-section of 100 × 100 μm square. This design pattern enables generating 20–level linear concentration gradients of the feeding solutions.

As shown in Figure 2B, the reactor arrays can carry out high throughput synthesis by dividing 100 reactors into five groups. That is, 100 wet-chemical reactions of different synthesis parameters with 20–level concentration gradients and 5–level temperature gradients can be held simultaneously. Each reactor has an inner diameter of 7 mm and a depth of 10 mm. That is, the maximum volume of each reactor is 0.38 mL. We chose PTFE as the material of this reactor arrays because PTFE can operate at high temperatures up to 260°C, and it is also chemically inert. We used 5 positive temperature coefficient (PTC) heating plates with different temperatures for each reactor group to achieve 5–level temperature gradients.

### Theoretical Calculation and Experimental Verification of the Platform

Theoretically, the microfluidic chip should be able to generate stable 20–level concentration gradients of the injected solutions only when the flow inside the chip is laminar according to the theory of fluid mechanics. To make sure the flow is laminar, the Reynolds number of the fluid should be smaller than 2100. Reynolds number can be expressed as follows (Young et al., 2000):

(1) $$\begin{array}{l} Re=\frac{\rho vD}{\mu } \end{array}$$

In which ρ is the density of the fluid, *v* is the velocity of the fluid, *D* is the diameter of the pipe/channel (100 μm in this case), and μ is the dynamic viscosity of the fluid. Accordingly, to ensure the flow is laminar inside the chip, the maximum fluid velocity in the channel is:

(2) $$\begin{array}{l} v_{max}\;=\;\frac{\mu {Re}_{max}}{\rho D} \end{array}$$

In which *Re*_*max*_ = 2100. Thus, the maximum flow rate inside the chip is:

(3) $$\begin{array}{l} Q_{max}\;=\;v_{max}\frac{\pi D^{2}}{4}\;=\;\frac{\pi {\mu DRe}_{max}}{4\rho } \end{array}$$

As the flow rates inside the chip and inside the syringe are equal, we can derive the maximum injection speed of the injection device:

(4) $$\begin{array}{l} u_{max}=\;Q_{max\;}\frac{4}{\pi d^{2}}\;=\;\frac{\mu {DRe}_{max}}{\rho d^{2}} \end{array}$$

In which *d* is the inner diameter of a typical 50 mL syringe (2.9 cm in this case). The detailed calculation results of *u*_*max*_ for water and propylene glycol are shown in Supplementary Table 1. According to these results, the flow is laminar if the injection speed is smaller than 10.7 mm s^−1^. Considering the maximum injection speed and the maximum torque of the stepping motor synthetically, the injection speed was set to be 0.1 mm s^−1^. In this way, we successfully derived the optimal settings of the injection device.

After the theoretical calculation, we conducted an inductively coupled plasma optical emission spectrometer (ICP–OES) test to see whether the chip can generate a stable 20–level concentration gradient profile as expected.

**Figure 3:**
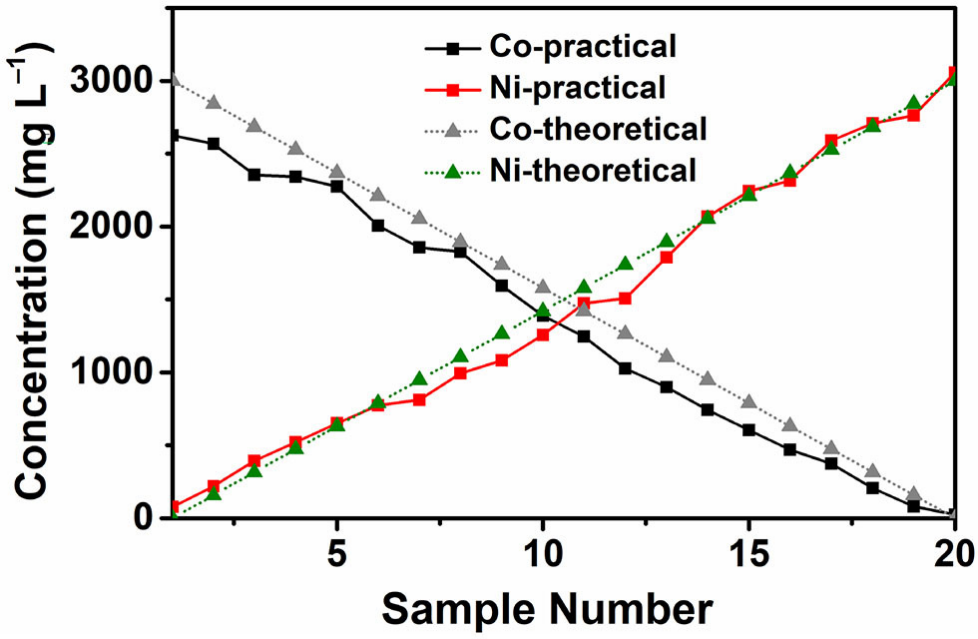
Concentration gradient test results with CoCl_2_ and NiCl_2_ water solutions using ICP–OES.

Figure 3 shows the ICP–OES test results and the theoretical concentration gradients generated by the microfluidic chip. 50.85 mM CoCl_2_ water solution and 50.85 mM NiCl_2_ water solution are used as two solutions and injected into the chip using the injection device. Then 20 solutions with different compositions are collected independently and tested by ICP–OES. The results show that the practical concentration is almost in a linear relationship with the sample number, which is close to the theoretical concentration. From the ICP–OES test results, we can conclude that the microfluidic chip can generate a stable 20–level concentration gradients of the two water solutions.

**Figure 4:**
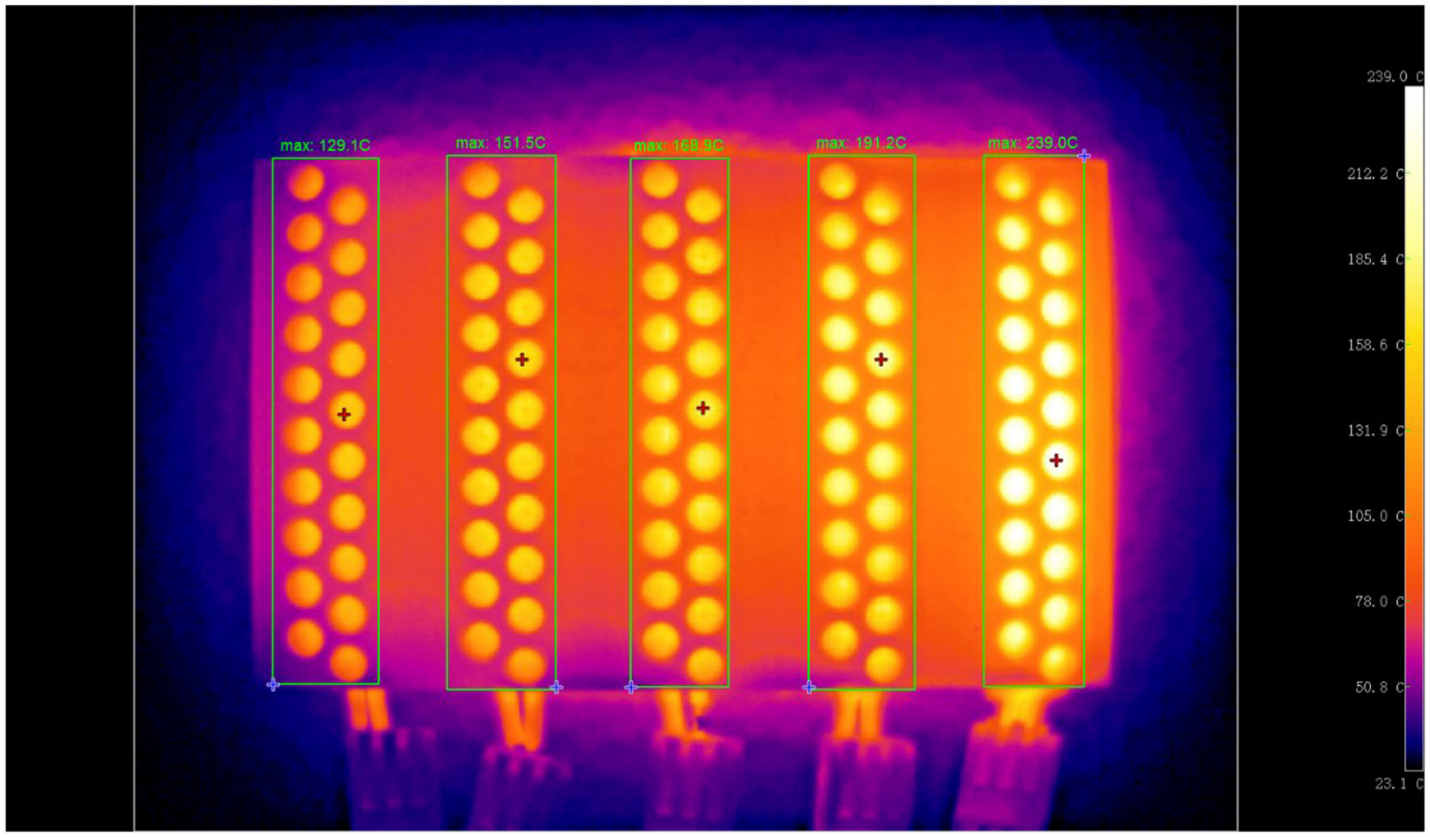
The infrared thermal image of the reactor arrays (at work).

Furthermore, to verify whether desired temperature gradients can be generated using the PTC heating plates attached to the reactor arrays, an infrared thermal image was captured and shown in Figure 4. Five groups of reactors can be heated at five different temperatures ranging from 120 to 230°C (for each PTC heating plate, the temperature error is ± 10°C). From this infrared thermal image, we can see that the five groups of reactors show different temperatures with a gradient pattern. In the five temperature regions surrounded by the rectangles, the highest temperatures are identified to be 129.1, 151.5, 168.9, 191.2, and 239.0°C, respectively. Thus, we can conclude that the reactor arrays meet the design standards and can generate stable 5–level temperature gradients as expected.

### Synthesis of the Bimetallic Powder Materials

In a typical synthesis procedure of Co–Ni bimetallic powder materials, 1 mmol cobalt (II) chloride hexahydrate (AR, Sinopharm Chemical Reagent Co., Ltd), 14 mmol NaOH (AR, XiLong Scientific Co., Ltd), and 0.5 mL pure water were dissolved in propylene glycol (AR, Sinopharm Chemical Reagent Co., Ltd) to make a 20 mL solution. Similarly, 1 mmol nickel (II) chloride hexahydrate (AR, Sinopharm Chemical Reagent Co., Ltd), 14 mmol NaOH, and 0.5 mL pure water were dissolved in propylene glycol to make another 20 mL solution (Guan et al., 2011). We chose propylene glycol as the organic solvent because it can serve as reductant and is also compatible with the material PDMS, according to previous studies (Lee et al., 2003). Both solutions were magnetically stirred for 2 h to make sure the solutions are uniform and transparent. This step is crucial because the large particles that existed in the reagents may cause jamming of the microfluidic chip with a channel width of 100 μm.

### Operation of the Microfluidic-Based Composition and Temperature Controlling Platform

After the preparation of the reagents, we can operate the microfluidic-based composition and temperature controlling platform to transport the reagents into the reactor arrays and start the HT synthesis. First, the reagents are introduced in two 50 mL syringes, respectively. Next, the automated injection device was set to run, generating reagent mixtures with 20–level concentration gradients into 20 centrifugal tubes. Afterward, all reactors were filled with desired reagents. Finally, the PTC heating plates attached to the reactor arrays were connected to a 220 V AC power supply to start the reaction process. To minimize solvent vaporization, we used a polyimide tape to seal the reactors. After a reaction time of 3 h, the PTC heating plates were unplugged so that the reactor arrays can be cooled down to room temperature naturally. To ensure that the PTC heating plates can work at designated temperature, the medium they contact should have low thermal conductivity.

All synthesized Co–Ni powders were further precipitated and isolated by a centrifugal machine. The products were washed with pure water mixed with ethanol for three times and dried in an oven at 80°C for 12 h.

### Material Characterization

All ICP–OES tests were conducted using an Agilent 720 ICP–OES system. The infrared thermal image was captured by a Magnity MAG30 on-line thermal imaging system. The surface morphologies were characterized by a Hitachi S−4800 field emission scanning electron microscope (FESEM). The elemental analysis was performed by an energy dispersive spectrometer (EDS) device attached to the FESEM. The X-ray photoelectron spectroscopy (XPS) was performed by Thermo SCIENTIFIC ESCALAB 250Xi with Al Kα X-rays as photon source (1486.8 eV).

## Results and Discussion

### Reagent Introduction Into the Reactor Arrays

**Figure 5:**
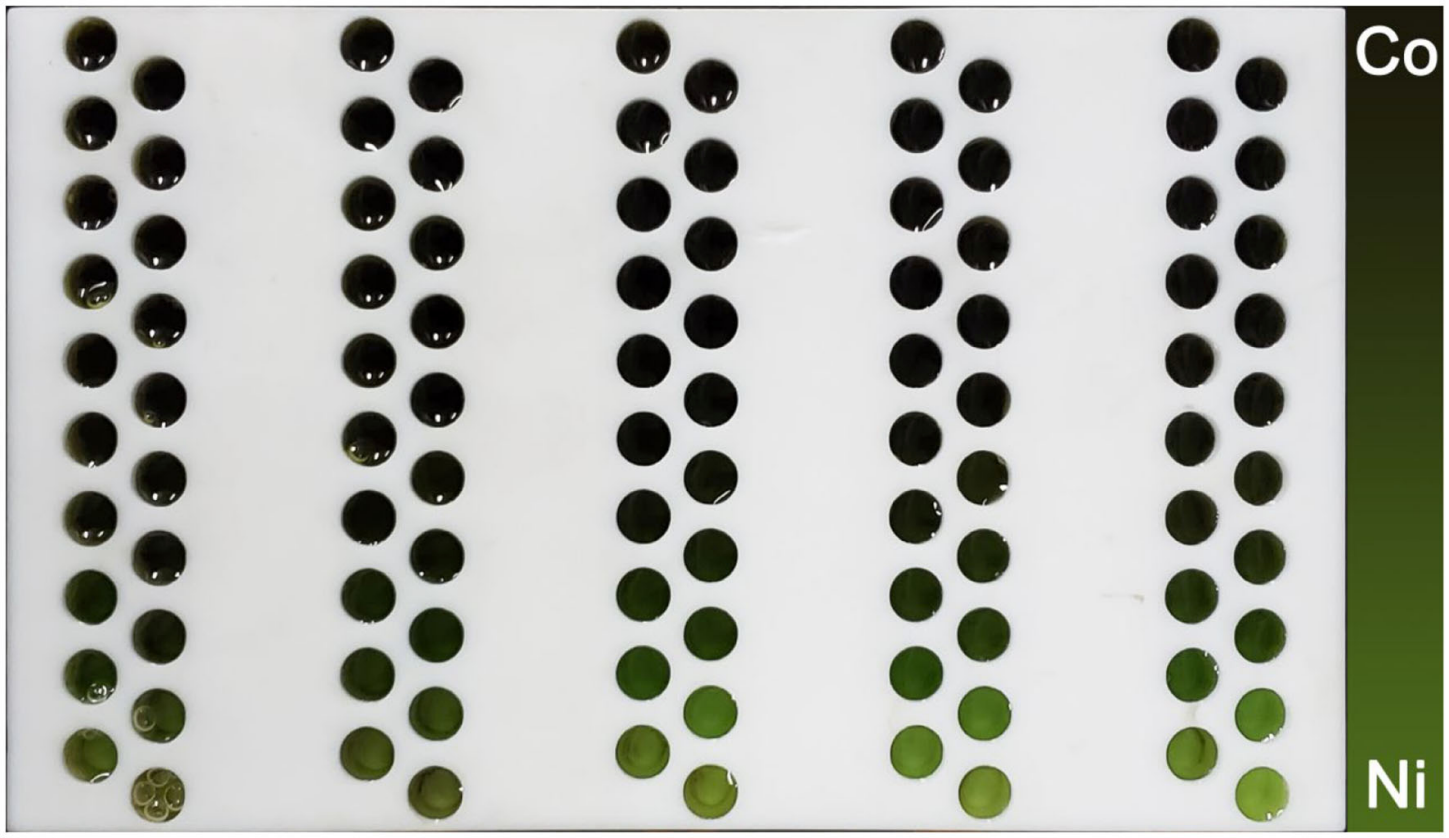
Photo of the reactor arrays before reaction, showing the concentration gradients of the Co–Ni reagents.

Figure 5 shows the photo of the reactor arrays after the introduction of Co–Ni reagents using the microfluidic-based composition and temperature controlling platform. In each group of reactors, the colors of the reagents follow a gradient pattern from one side to the other side. According to the concentration gradients generated by the microfluidic chip (shown in Figure 3), we can see that the reactors closer to the Co side have a higher content of Co element in the reagents and vice versa. This result reveals that reagents with desirable concentration gradients can be successfully generated and introduced into the reactor arrays using the platform.

### Determination of Minimum Temperature Required for the Reaction System

**Figure 6:**
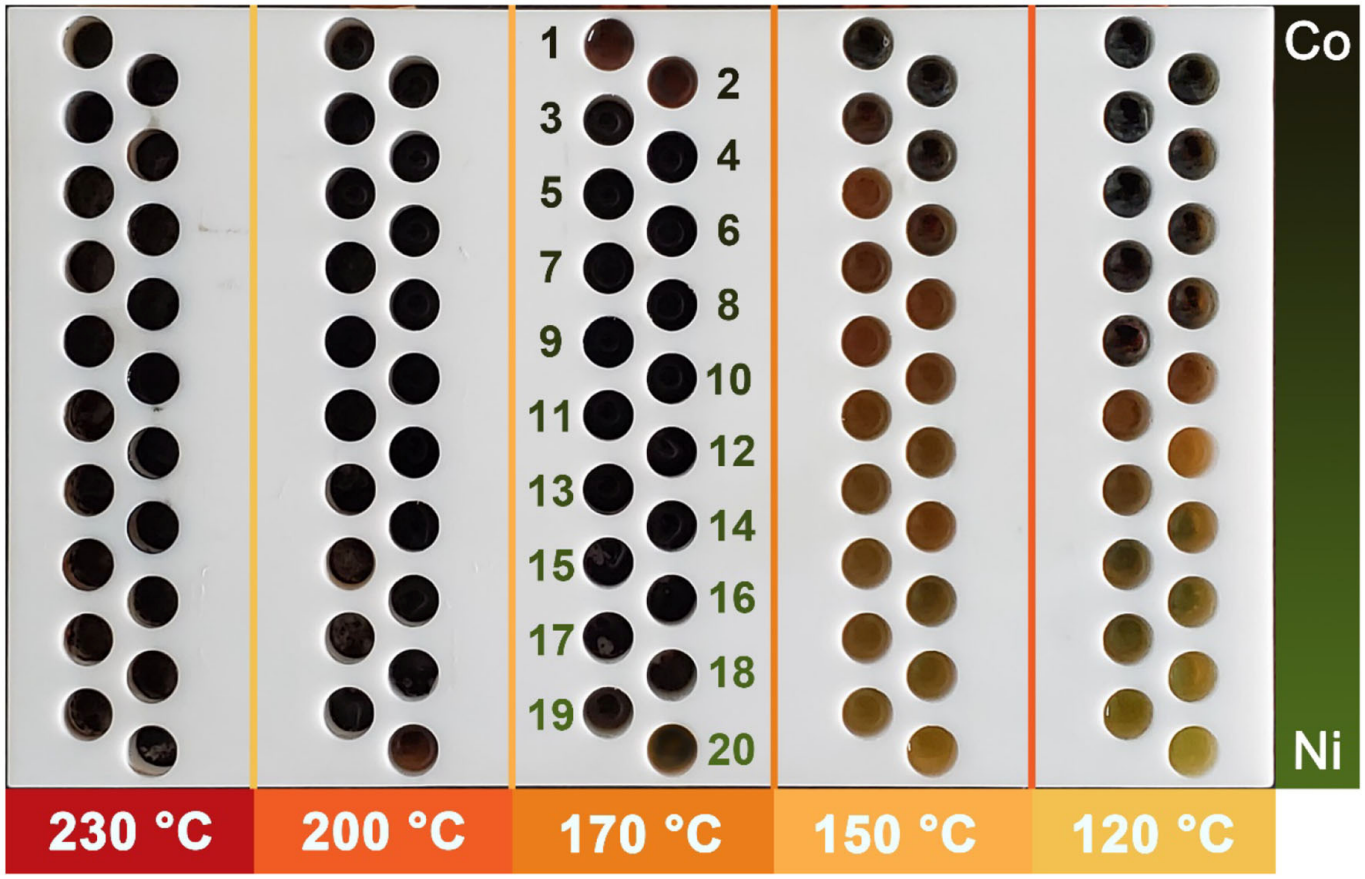
Photos of the reactor arrays after synthesis of Co–Ni bimetallic powder materials. The numbers 1–20 in the 170°C region indicate the label of each individual reactor.

Figure 6 shows the reactor arrays after the wet-chemical synthesis of Co–Ni bimetallic powder materials. According to our preliminary test results, the reactor in which the powders were successfully synthesized should show a black color. From Figure 6, most reactors in 120 and 150°C regions are not black, indicating unsuccessful synthesis in these locations. While in 170, 200, and 230°C regions, all reactors show black color. According to this observation result, we picked the samples synthesized in three high-temperature regions for further characterization (60 samples in total, 20 samples in each temperature group). This result indicates that temperatures above 150°C are required for the synthesis of Co–Ni bimetallic powder materials in propylene glycol, and temperatures above 170°C ensure successful synthesis. According to some previous researches, common synthesis temperatures for polyol reaction systems with traditional wet-chemical approaches are 150°C (Liu et al., 2008), 160°C (Guo et al., 2018), and 170°C (Liu et al., 2009), which are consistent with our conclusion. This result proves that our platform is highly advantageous for one-step determination of the minimum temperature required for wet-chemical reaction systems.

### Morphology and Elemental Characterization Results of Synthesized Co–Ni Bimetallic Powder Materials

The synthesized 60 samples of Co–Ni bimetallic powder materials were characterized by SEM and EDS. The EDS data was collected from the same region of the samples with the corresponding SEM images. The detailed mass fraction data from the EDS characterization results can be found in Supplementary Table 2. According to the EDS results, all 60 samples only contain element Co, Ni, and O. The sample numbers are consistent with the reactor labels shown in Figure 6. Theoretically, a smaller sample number indicates lower Ni content and higher Co content, according to the concentration gradients confirmed by the ICP–OES test results shown in Figure 3.

**Figure 7:**
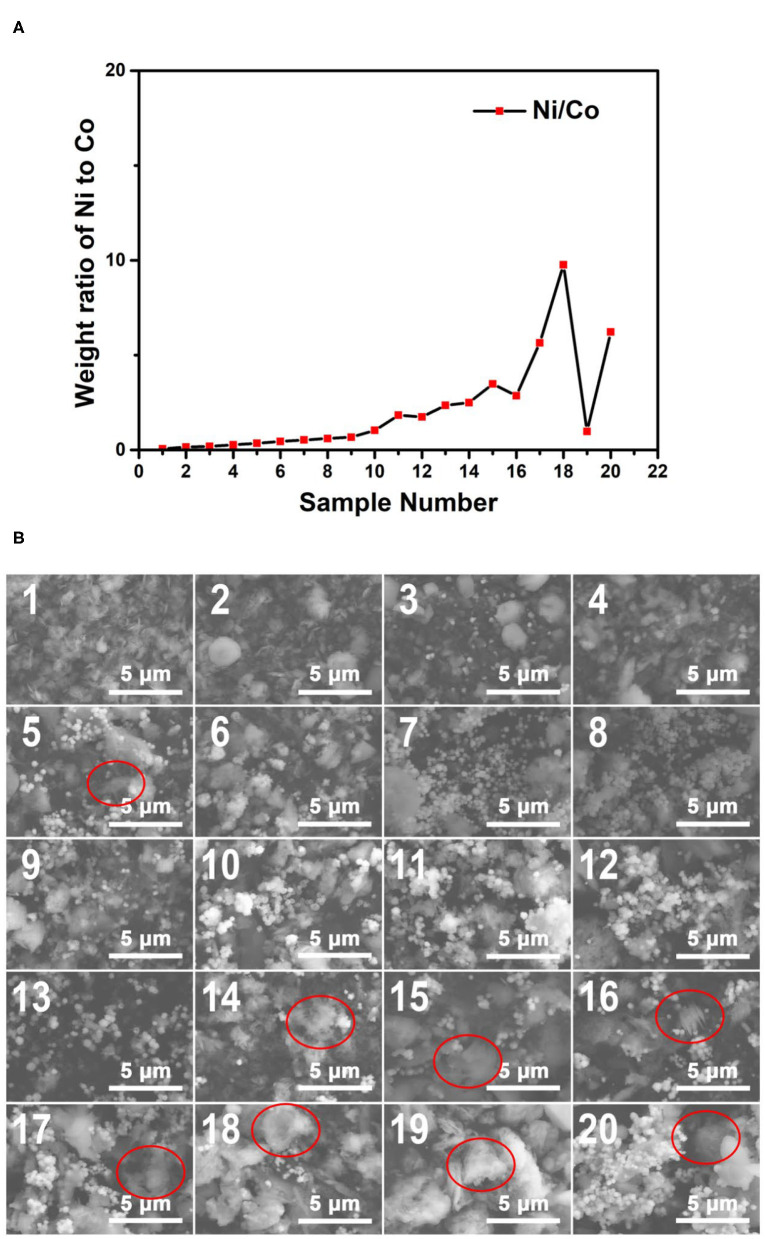
**(A)** The weight ratio plot of Ni to Co of the obtained samples synthesized at 170°C; **(B)** The SEM images of the obtained samples synthesized at 170°C. The EDS spectra of the samples corresponding to the SEM characterization regions are shown in Supplementary Figure 2.

The plot of the weight ratio data of Ni to Co from the EDS results of the 170°C samples is shown in Figure 7A. It can be concluded that with the increase of the sample number, the Ni/Co weight ratios increase in general. Indeed, some of the samples do not perfectly comply with this rule. This phenomenon may result from the ununiform distribution of the particles. The EDS spectra also showed this tendency (insets in Figure 7B). The EDS results confirm that the concentration gradients of reagents have successfully transformed into composition gradients of the 170°C reaction products, proving the functionality of our microfluidic-based composition and temperature controlling platform.

Figure 7B shows the SEM images of the Co–Ni bimetallic powder materials synthesized at the reaction temperature of 170°C. It can be seen clearly from the SEM images that samples Nos. 7–13 show the characteristic morphology of small particles (~0.5 μm), which can barely be identified in other samples in the same group. From Supplementary Table 2, we can see that the weight ratios of Ni to Co in samples Nos. 7–13 are relatively close to 1:1. This indicates that the small particles tend to form under an intermediate weight ratio of Co and Ni under the temperature of 170°C. Meanwhile, floc morphologies can be observed in most of the samples (marked with red circles). This is due to the relatively slow crystallization activities restricted by this reaction temperature. Particularly, the samples with larger sample numbers (higher Ni/Co weight ratios) tend to show more floc features. Then we can come to a preliminary conclusion that products with higher Ni/Co weight ratios may require a higher temperature to obtain particle morphologies. These SEM characterization results show that the Co–Ni bimetallic powder materials can be synthesized at 170°C, and the products contain both floc and particle morphologies.

**Figure 8:**
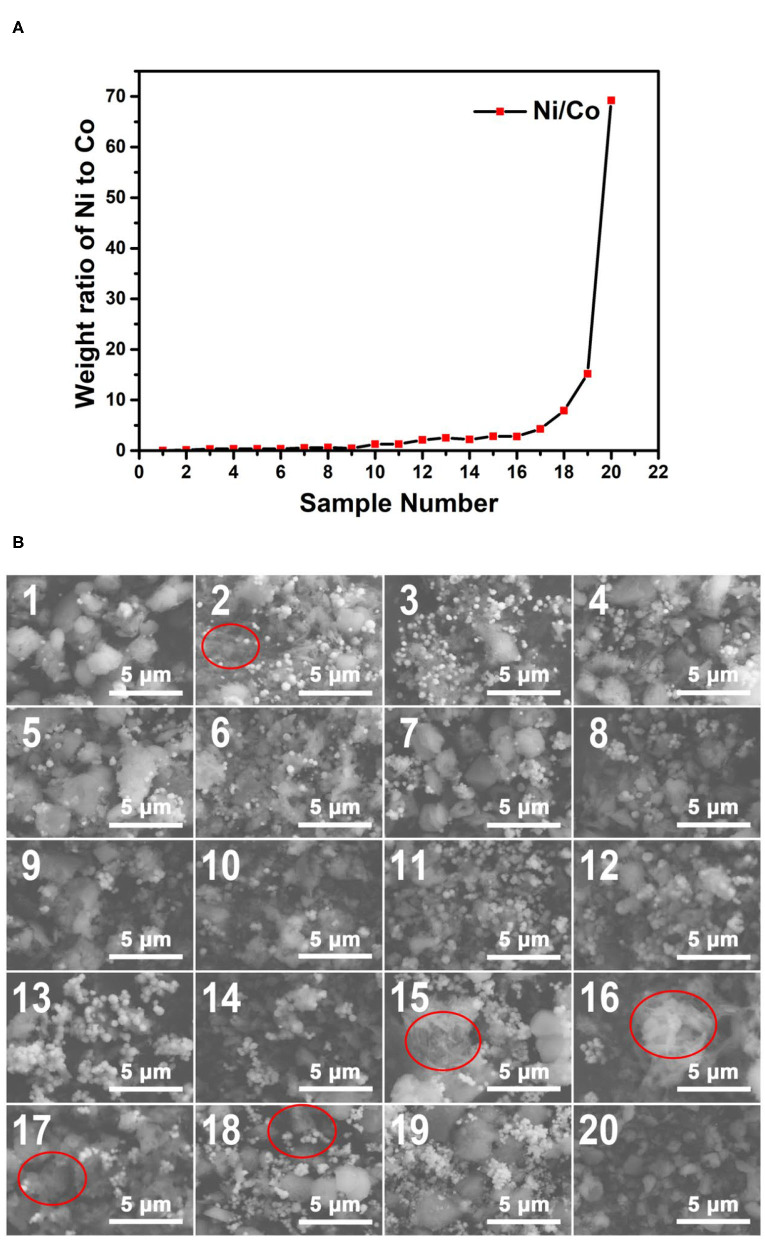
**(A)** The weight ratio plot of Ni to Co of the obtained samples synthesized at 200°C. **(B)** The SEM images of the obtained samples synthesized at 200°C. The EDS spectra of the samples corresponding to the SEM characterization regions are shown in Supplementary Figure 3.

The plot of the weight ratio data of Ni to Co from the EDS results of the 200°C samples is shown in Figure 8A. Like the results of the 170°C samples, with the increase of the sample number, the Ni/Co weight ratios increase with almost no exception. This tendency can be further confirmed by the EDS spectra shown as insets in Figure 8B. These results indicate that a wide range of composition gradients of the 200°C reaction products were successfully formed using our microfluidic-based composition and temperature controlling platform.

Figure 8B shows the SEM images of the Co–Ni bimetallic powder materials synthesized at the reaction temperature of 200°C. Apart from small particles (~0.5 μm), large particles (~2 μm) appear in most of the samples. This phenomenon indicates that with the increase of reaction temperature, the particle size also increases since the nucleation and growth process benefit from higher temperatures. Another interesting phenomenon is that some 200°C samples (those with high Ni/Co weight ratios) still show floc morphologies (marked with red circles). This result proves that our preliminary conclusion (products with higher Ni/Co weight ratios may require a higher temperature to obtain particle morphologies) is still correct for the 200°C samples. The SEM characterization results prove that the 200°C samples have more particle morphologies and larger particle size than the 170°C samples.

**Figure 9:**
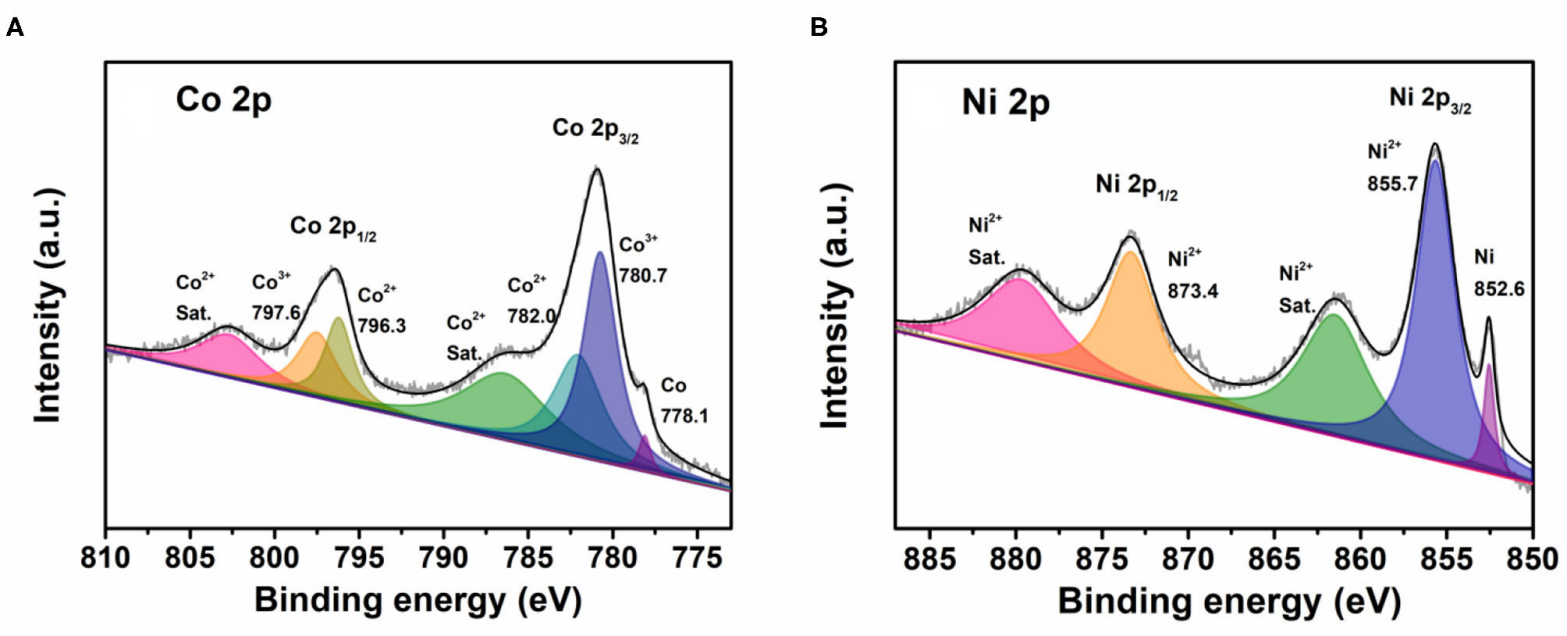
The XPS spectra of sample No. 11: **(A)** The Co 2p spectrum; **(B)** The Ni 2p spectrum.

To further confirm the oxidation state of the Co–Ni bimetallic powder materials synthesized with our platform, we picked sample No. 11 to perform XPS analysis. Figure 9A shows the Co 2p spectrum, in which three Co species are revealed in the sample. More specifically, the peaks at 780.7 and 797.6 eV are attributed to Co^3+^ species and the peaks at 782.0 and 796.3 eV are assigned to Co^2+^ species. Moreover, the peak at 778.1 eV confirms the existence of metallic cobalt. Figure 9B shows the Ni 2p spectrum and it reveals two Ni species in the sample. The peaks at 855.7 and 873.4 eV can be attributed to Ni^2+^ species and the peak at 852.6 eV proves the existence of metallic nickel. The XPS characterization results show that the precursors containing ionic cobalt and nickel can be reduced to metallic cobalt and nickel, while some of the metals are oxidized because of the semi-open reaction condition.

**Figure 10:**
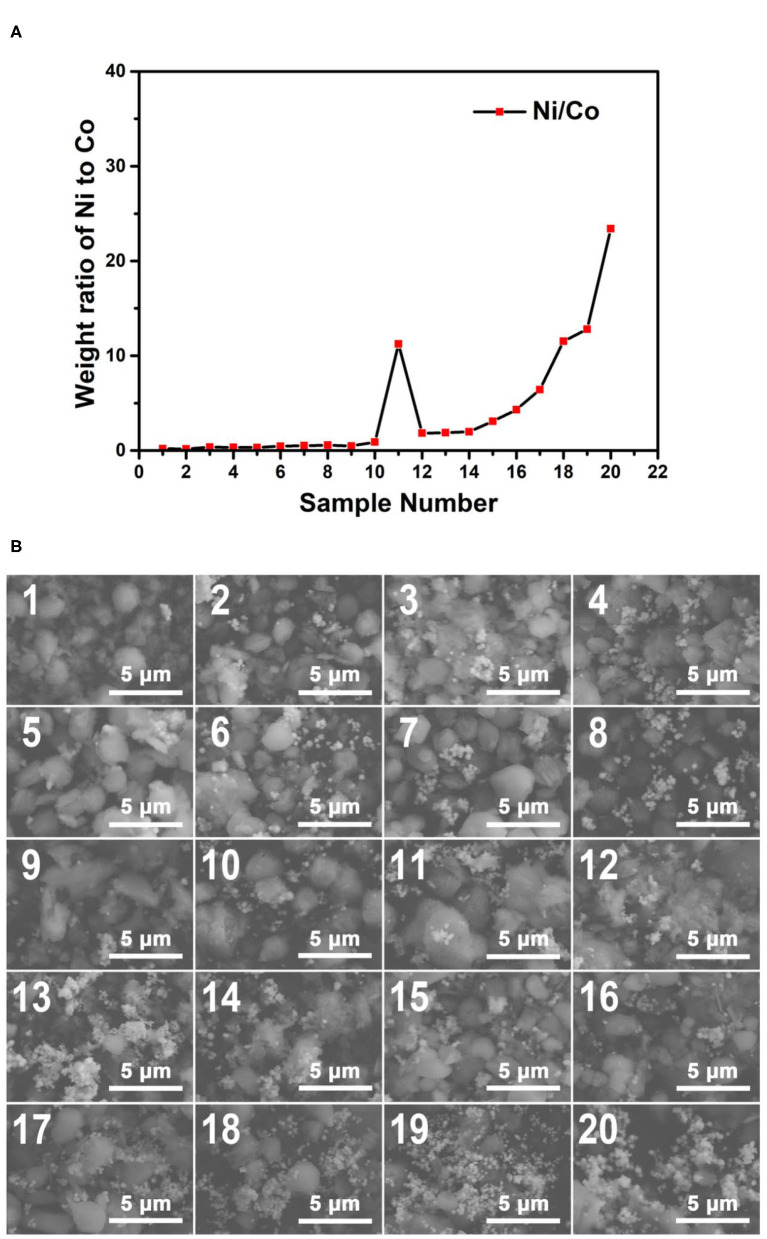
**(A)** The weight ratio plot of Ni to Co of the obtained samples synthesized at 230°C; **(B)** The SEM images of the obtained samples synthesized at 230°C. The EDS spectra of the samples corresponding to the SEM characterization regions are shown in Supplementary Figure 4.

The plot of the weight ratio data of Ni to Co from the EDS results of the 230°C samples is shown in Figure 10A. Among the 230°C samples, No. 11 showed an irregular pattern due to the ununiform distribution of the particles. Except for sample No. 11, as the sample number increases, the weight ratios of Ni to Co increase in general. The EDS spectra shown as insets in Figure 10B is consistent with this tendency. These results confirm that the 230°C reaction products also have the concentration gradients as we expected.

Figure 10B shows the SEM images of the Co–Ni bimetallic powder materials synthesized at the reaction temperature of 230°C. The overall morphologies of the 230°C samples are more regular and more uniformly distributed. In these samples, the morphologies of large particles (~2 μm) become dominant, making them distinct from the 170°C samples and the 200°C samples. Furthermore, floc morphologies are almost absent in all 230°C samples. This result proves that the reaction temperature of 230°C can result in desired particle morphologies for the Co–Ni bimetallic powder materials.

In summary, the discussion of SEM and EDS characterization results can be expressed as follows: First, according to the EDS results, with the increase of sample number, the weight ratios of Ni to Co increase in general; Second, according to the SEM images, with the increase of reaction temperature (from 170 to 230°C), the floc morphologies in the samples gradually change into particle morphologies, and the particle size increases; Third, when the Ni/Co weight ratios of the samples are high, the formation of particle morphologies requires higher temperature with given reaction time. These results prove that our platform is capable of HT wet-chemical synthesis with controllable product composition. Moreover, these results provide valuable guidance for further optimization of the synthesis parameters of this wet-chemical system, proving the practicality of our microfluidic-based composition and temperature controlling platform.

## Conclusion

In this paper, we successfully prepared a microfluidic-based composition and temperature controlling platform toward facile HT wet-chemical synthesis. The microfluidic chip is designed to generate stable 20–level concentration gradients of the reagents. The reactor arrays containing 100 reactors are designed to carry out wet-chemical synthesis with the parameters of 20 concentration gradients and 5 temperature gradients, that is, 100 different reaction conditions. We successfully synthesized Co–Ni bimetallic powder materials using this platform in a facile and automated workflow. According to the EDS characterization results, the samples in each temperature group showed a gradient of elemental composition as expected. According to the SEM characterization results of the samples, the morphologies of the obtained Co–Ni bimetallic powder materials were highly relevant to the reagent concentrations and reaction temperatures. All these results show that our microfluidic-based composition and temperature controlling platform is ideal for preliminary experiments aimed at investigating the wet-chemical synthesis parameters. Moreover, the platform has the flexibility to fulfill the need for parameter investigations on a larger scale. The application of this HT platform can be further extended to most wet-chemical reactions under aqueous and non-aqueous conditions.

The main contributions of the microfluidic-based composition and temperature controlling platform can be concluded as: First, the platform enables one-step determination of the minimum temperature required for a wet-chemical reaction system. Second, the platform provides valuable information for the interrelationships of composition, temperature, and morphology of the wet-chemical reaction products. Third, the platform provides a significant increase in efficiency compared with the traditional wet-chemical approach. The composition and temperature controlling platform points out a new way of introducing microfluidic techniques into HT synthesis, enabling timesaving, environmentally friendly, and cost-effective wet-chemical synthesis and parameter investigation in a facile and automated workflow.

## Data Availability Statement

The raw data supporting the conclusions of this article will be made available by the authors, without undue reservation.

## Author Contributions

YH, BL, YW, WH, and CZ contributed to conception and design of the study. YH and BL completed the design and fabrication of the platform. XL and ML performed the SEM and EDS analysis. JD, XH, and YD performed the statistical analysis. YH wrote the first draft of the manuscript. All authors contributed to manuscript revision, read, and approved the submitted version.

## Conflict of Interest

The authors declare that the research was conducted in the absence of any commercial or financial relationships that could be construed as a potential conflict of interest.
