# Supplementary material for: Facile High Throughput Wet-Chemical Synthesis Approach Using a Microfluidic-Based Composition and Temperature Controlling Platform
Source: Front Chem. 2020 Nov 2;8:579828. doi: 10.3389/fchem.2020.579828 (PMC7667272; doi:10.3389/fchem.2020.579828)
Supplement: Supplementary file 3 [file Data_Sheet_1.pdf]

frontiers

Supplementary Material

Facile High Throughput Wet-Chemical Synthesis Approach Using a Microfluidic-based Composition and Temperature Controlling Platform

Yang Hu, Bin Liu, Yating Wu, Ming Li, Xiaorui Liu, Jia Ding, Xiaopeng Han, Yida Deng, Cheng Zhong*, Wenbin Hu

Contents:

1. Figure S1. Real photo of the silicon mold.

2. Figure S2. The EDS spectra of the 170 °C samples.

3. Figure S3. The EDS spectra of the 200 °C samples.

4. Figure S4. The EDS spectra of the 230 °C samples.

5. Table S1. Calculation results of u_{max} for water and propylene glycol.

6. Table S2. Mass fraction of Co and Ni in Co–Ni bimetallic powder materials synthesized at different temperatures (%).

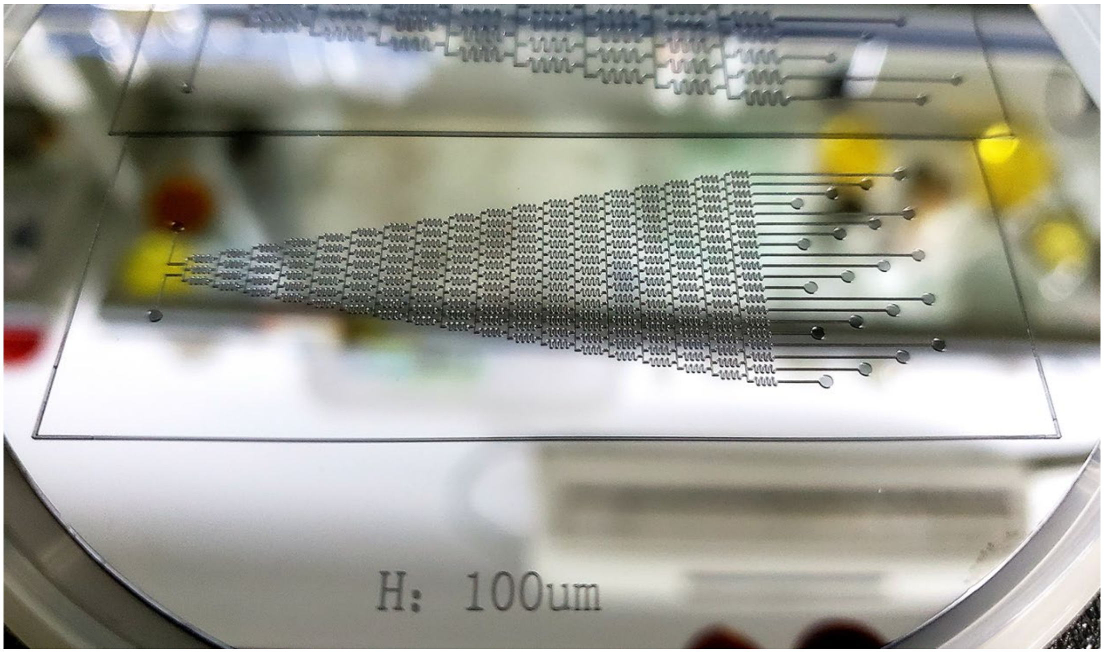

Fig. S1. Real photo of the silicon mold, showing a Christmas tree-like structure. The size of the micro tunnels is 100 μm.

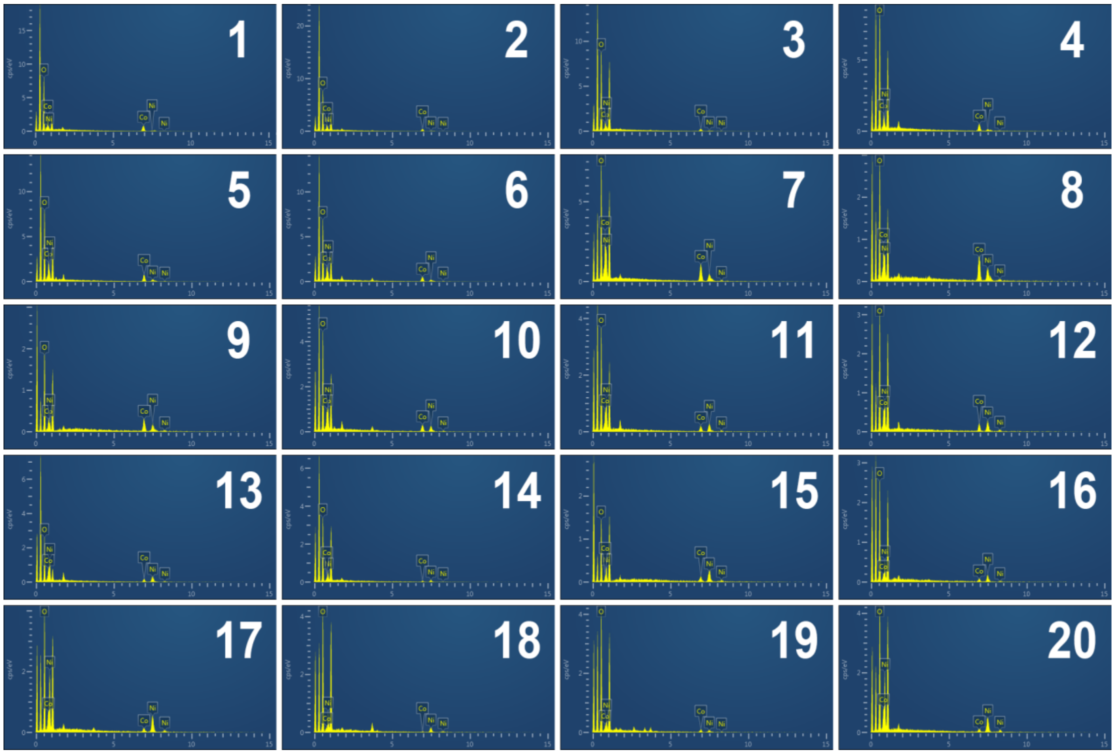

Fig. S2. The EDS spectra of the 170 °C samples.

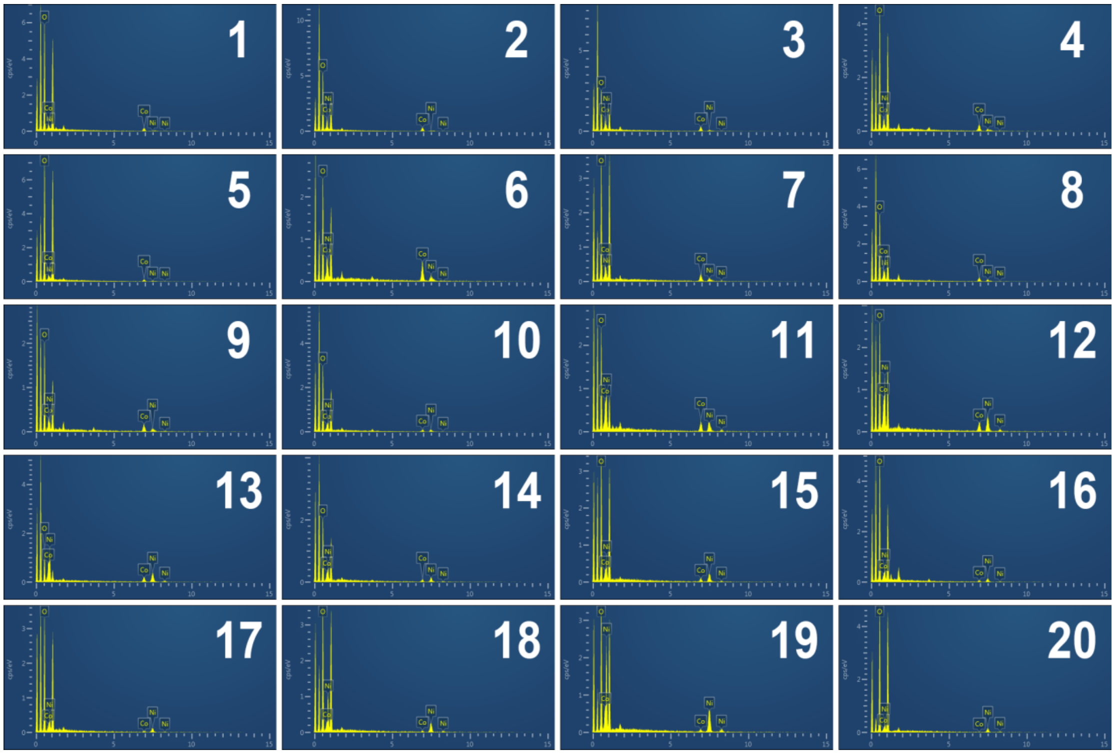

Fig. S3. The EDS spectra of the 200 °C samples.

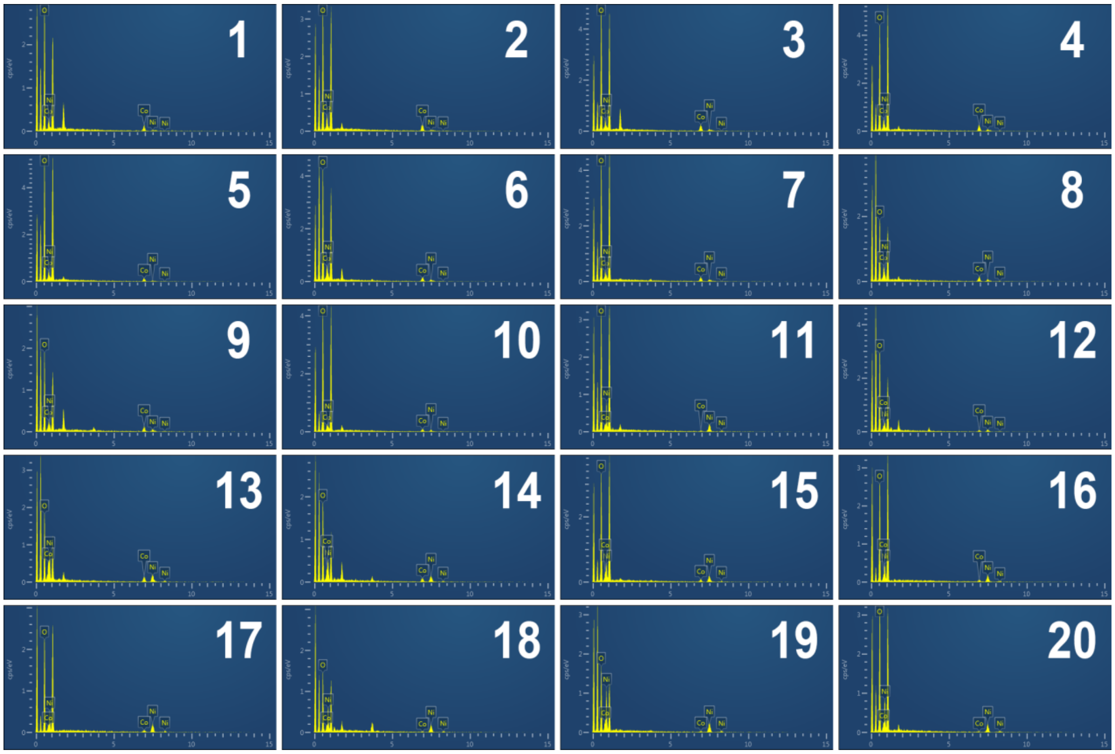

Fig. S4. The EDS spectra of the 230 °C samples.

Table S1. Calculation results of u_{max} for water and propylene glycol (Tanaka et al., 1988)

System	ρ (g cm^{-3})*	μ (g cm^{-1} s^{-1})*	u_{max} (mm s^{-1})**
Water	0.997	0.0089	0.2227
Propylene glycol	1.036	0.4439	10.70

* The density (ρ) and viscosity (μ) data used for calculation are values at 298.15 K (25 °C).

** The u_{max} data were calculated using Equation (4) of the manuscript.

In which $Re_{max} = 2100$, $D = 100$ μm, $d = 2.9$ cm.

Table S2. Mass fraction of Co and Ni in Co–Ni bimetallic powder materials synthesized at different temperatures (%)

Temp. / No.	170 ℃		200 ℃		230 ℃	
	Co	Ni	Co	Ni	Co	Ni
1	47.73	2.80	25.77	0.00	28.69	5.76
2	33.95	5.13	36.52	5.17	32.70	5.28
3	22.69	4.21	42.08	13.93	29.41	10.27
4	33.03	9.18	29.95	11.53	31.67	10.96
5	39.42	14.19	15.14	5.43	20.86	6.67
6	35.14	15.68	47.27	17.98	25.36	11.50
7	41.89	22.27	29.59	16.56	22.36	11.66
8	45.45	27.60	28.58	17.62	31.89	18.00
9	39.95	27.01	34.54	16.37	32.15	15.67
10	26.61	27.59	17.22	22.30	15.52	13.89
11	19.91	36.55	28.05	36.74	3.55	39.91
12	21.86	38.14	21.59	46.01	11.80	21.64
13	19.26	45.42	22.44	56.30	21.83	41.04
14	10.05	25.06	16.17	35.55	17.88	35.50
15	15.64	54.41	12.96	36.98	11.17	34.37
16	13.06	37.42	8.78	24.52	8.91	38.40
17	9.30	52.53	6.63	28.53	7.03	45.18
18	3.18	31.05	5.55	43.82	4.61	53.20
19	16.08	15.83	4.10	62.25	4.46	57.06
20	8.35	51.96	0.43	29.76	1.76	41.23

References

Tanaka, Y., Ohta, K., Kubota, H., and Makita, T. (1988). Viscosity of aqueous solutions of 1,2-ethanediol and 1,2-propanediol under high pressures. *Int. J. Thermophys.* 9, 511-523. doi: 10.1007/BF00503150
